# PD35 A Decade In Review: Assessing The Quality Of Clinical Evidence For Oncology Drugs Submitted To A National Health Technology Assessment Agency

**DOI:** 10.1017/S0266462324002988

**Published:** 2025-01-07

**Authors:** Heather Eames, Joy Leahy, Lea Trela-larsen, Laura McCullagh

## Abstract

**Introduction:**

To ensure accurate and objective comparisons between interventions, the best available clinical evidence must be used. This updated analysis sought to review the quality of clinical evidence submitted by applicant companies to a national health technology assessment (HTA) agency in Ireland. This analysis aimed to assess the association between the quality of clinical evidence and the reimbursement recommendation.

**Methods:**

A retrospective analysis of the clinical evidence supporting all oncology HTAs submitted to the National Centre for Pharmacoeconomics (NCPE) from May 2012 to December 2023 was conducted. NCPE recommendations were classed as “conditional-positive/positive” or “conditional-negative/negative” for the purposes of this analysis. Data extraction of key clinical evidence characteristics relating to the main clinical trial and indirect treatment comparison (ITC), if applicable, was informed by EUnetHTA guidance. Trends in clinical evidence characteristics were assessed over time. Potential associations between the quality of clinical evidence and reimbursement recommendations were investigated using a hierarchy of chi-squared tests and multivariate regression analysis.

**Results:**

This analysis included 101 completed HTAs, 84 of which were supported by traditional randomized controlled trials (RCTs). The proportion of HTAs supported by single-arm trials (SATs) increased to 55.6 percent in 2023. HTAs supported by RCTs were more likely to receive a “conditional-positive/positive” recommendation, compared with those supported by SATs (42.8% versus 17.6%). Most of the RCTs were open-label studies (n=49), however recommendations were similar among HTAs supported by blinded and open-label studies. The overall survival data maturity was variable. ITCs were required in 76 HTAs, mostly supported by anchored ITCs. SATs supported by unanchored ITCs were more likely to receive a “conditional-positive/positive” recommendation than naïve comparisons.

**Conclusions:**

Reliance on SATs and open-label studies has increased over the past decade. This analysis proposes that HTAs supported by RCTs remain the gold standard for HTA. A key limitation of this study is that the quality of evidence submitted is only one domain of the HTA evaluation process. No tests were statistically significant, which was partly explained by the small sample sizes.

